# Effects of Defoliation at Fruit Set on Vine Physiology and Berry Composition in Cabernet Sauvignon Grapevines

**DOI:** 10.3390/plants10061183

**Published:** 2021-06-10

**Authors:** Eleonora Cataldo, Linda Salvi, Francesca Paoli, Maddalena Fucile, Giovan Battista Mattii

**Affiliations:** Department of Agriculture, Food, Environment and Forestry (DAGRI), University of Florence, 50019 Sesto Fiorentino, Italy; eleonora.cataldo@unifi.it (E.C.); linda.salvi@unifi.it (L.S.); francesca.paoli@unifi.it (F.P.); maddalena.fucile@unifi.it (M.F.)

**Keywords:** leaf removal, defoliation, berry temperature, sugar content, anthocyanin content

## Abstract

Grapevine canopy defoliation is a fundamentally important technique for the productivity and quality of grapes. Leaf removal is a pivotal operation on high-density vines which aims to improve air circulation, light exposure, and leaf gas exchange. The effects of leaf removal (LR) on vine physiology and berry composition in Cabernet Sauvignon grapevines were studied during the 2018–2019 growing season in the Bolgheri area, Tuscany, Italy. The basal leaves were removed at fruit set at two severity levels (removal of four basal leaves of each shoot (LR4) and removal of eight basal leaves (LR8)). The two treatments were compared with the not defoliated control (CTRL). The following physiological parameters of vines were measured: leaf gas exchange, leaf water potential, chlorophyll fluorescence and indirect chlorophyll content. The results showed that defoliation increased single leaf photosynthesis. In addition, qualitative grape parameters (phenolic and technological analyses) and daytime and night-time berry temperature were studied. The results showed that leaf removal had an impact on total soluble solids (°Brix), titratable acidity, and pH. The LR8-treated grapes had higher titratable acidity, while those in the LR4 treatment had higher °Brix and extractable anthocyanin and polyphenol content. Berry weight was not significantly influenced by the timing and severity of basal defoliation. Therefore, this research aims to investigate the effects of defoliation at the fruit set on vines performance.

## 1. Introduction

Defoliation in the fruiting zone of grapevines is a classical canopy management practice in vineyards applied from spring until the end of summer [1]. Improvement of the microclimate after defoliation is mainly attributed to the increase in bunch temperature and modification of the shadow-light ratio [2,3,4]. Furthermore, this practice improves berry quality (for instance, pigmentation, aromatic compounds, and secondary metabolites), whereas it reduces conditions favorable to bunch rot complex diseases [5,6,7]. Even though leaf removal is more often applied to vigorous vines or in chilly climates where the environmental conditions and cluster microclimate are often inadequate to ensure adequate berry ripening [8], this agronomic practice is widely used, even in warmer climates and on plants that are not excessively vigorous to improve their performance. However, defoliation must be undertaken with care to prevent the risk of berry overheating and burning [9]. To obtain a high-quality grape and wine, many authors highlight the importance of achieving and maintaining the optimal ratio between grape yield and assimilation area [10,11,12]. In fact, quality viticulture is correlated to a balance between plant and agronomic management [13] and must address the match between reproductive organs and vegetative sink activity.

The effect of defoliation depends on the number of removed leaves, timing, grape variety, and climate [14]. Depending on the phenological stage, defoliation can be performed early (at pre-flowering), medium (at fruit set) or late (pre- or post-veraison). Early defoliation decreases grape yield by decreasing berry number and thus, cluster weight, whereas leaf removal during or after veraison impacts the primary and secondary synthesis of metabolites [15]. Nevertheless, some studies have shown that early leaf thinning can improve grape and wine quality. Defoliation before flowering was found to decrease fruit set and reduce grape yield of many varieties (e.g., Sangiovese, Merlot, Tempranillo), whereas under different microclimatic conditions, it increased anthocyanin and phenolic concentrations [6,16,17,18,19,20]. Moreover, the effects of leaf removal on yield also depend on severity [21,22,23]. Since carbohydrate supply at anthesis is a fundamental determinant of fruit set, leaf removal three or four weeks before anthesis normally reduces cluster compactness, yield, and total amount of sugar per berry [24]. On the other hand, if leaves are removed later in the phenological season (e.g., veraison) or fewer are removed (lower severity), yield is not significantly affected and it can even increase compared with non-defoliated treatments [25]. However, pre-flowering defoliation presents some risks as removing most of the photosynthetically active foliage when inflorescences have high C and N requirements forces the vine to further rely on reserves in roots and wood (competition for assimilates between reproductive and vegetative organs) [26,27]. Percival et al. [28] noted that with pre-flowering defoliation and the consequent increased exposure to solar radiation, a thicker layer of cuticular waxes was formed and correlated this thickening with increased resistance to cluster rot [29].

Fruit set defoliation is an efficient technique to reduce cluster compactness, to improve fruit composition, and control crop load in cultivars characterized by large berry size and improve their health [30].

Defoliation after flowering and at fruit set (compared with leaf removal at veraison) of all basal leaves up to the first cluster increases not only monoterpen content (especially nerol, linalool, and geraniol) but also alcohol, and decreases the concentration of volatile esters in white berries [31,32,33,34]. Conversely, shading in the canopy has implications for red berry alterations: an excessively shaded canopy microclimate can cause increased titratable acidity (malic and tartaric acids) and juice NH_4_-N and K content [35]. Changes in berry composition due to shade can be associated with known effects of the phytochrome on enzyme activity (including malic enzyme, phenylalanine ammonia lyase, invertase, PEP-carboxylase, and malic dehydrogenase) [4,36,37].

Many authors have reported balancing berry physiology ripening between technological and phenolic maturation, decreasing the accumulation of sugar, and manipulating the relationship between leaf area and yield by intervening at different phenological stages [38,39,40,41]. Among these, post-veraison defoliation seems to be the most promising. Poni [42] and Filippetti [43] reported that late defoliation, removing six or seven basal leaves and some lateral shoots from the upper two-thirds of the canopy, in Sangiovese cultivar caused sugar accumulation (at about 12 °Brix) to decrease without negatively affecting the concentration of anthocyanin and phenolic compounds. Nevertheless, leaf thinning can also affect cluster quality negatively. In fact, an excessive defoliation can lead to overexposed fruit (subjecting the berries to high temperature and high light intensity) and, in red cultivars, can reduce anthocyanin content [44].

The present investigation examines the effects of defoliation at fruit set on cv. Cabernet Sauvignon in local Mediterranean conditions with attention to the alternative severity of defoliation, especially to its effects on ecophysiological parameters, yield reduction, sugar and anthocyanin content. 

## 2. Results

### 2.1. Climatic Conditions 

**Figure 1:**
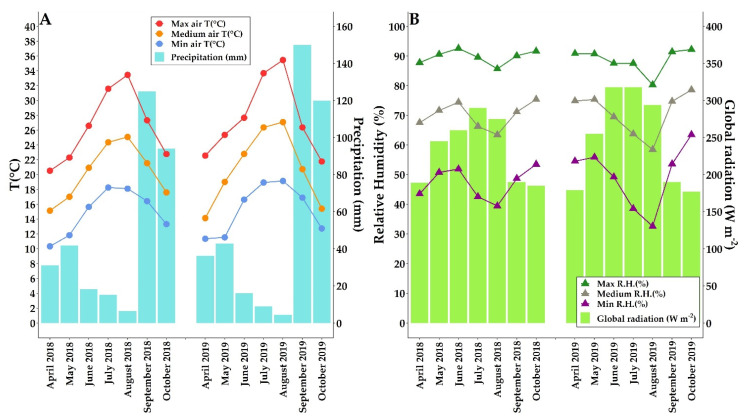
Climatic conditions of the experiment location. Monthly averages of mean, maximum, minimum air temperature (°C) and total monthly rainfall (mm) (**A**) were measured from April to October (2018–2019); monthly averages of mean, maximum, minimum relative humidity (%), and average monthly global radiation (RAD, W m^−2^) (**B**) were measured from April to October (2018–2019).

Temperature conditions were those typical of warm-Mediterranean viticulture climates, as reported in Figure 1. 

Rainfall throughout the season, April to October, was relatively higher in 2019 with 378.4 mm recorded, while during the berry ripening period (from June to August), 2019 turned out to be drier: 29.4 mm compared to 39.8 mm in 2018. The average air temperature, from April to October, was 20.2 °C in 2018 and 20.8 °C in 2019. The absolute maximum air temperature during the investigated period ranged from 33.4 °C in August 2018 to 35.4 °C in August 2019.

### 2.2. Leaf Gas Exchanges, Leaf Area, Leaf Water Potential, Leaf Chlorophyll a Fluorescence and Content

The total leaf area (m^2^) is presented in Table 1. The highest leaf area was observed in the control and the lowest in the LR8 treatment. All differences among the values were significant. The first measurement concerned the relief immediately after defoliation at the time of fruit set (18 June 2018 and 12 June 2019). The F-test showed significant effects of the two removals in comparison to the control treatment.

**Figure 2:**
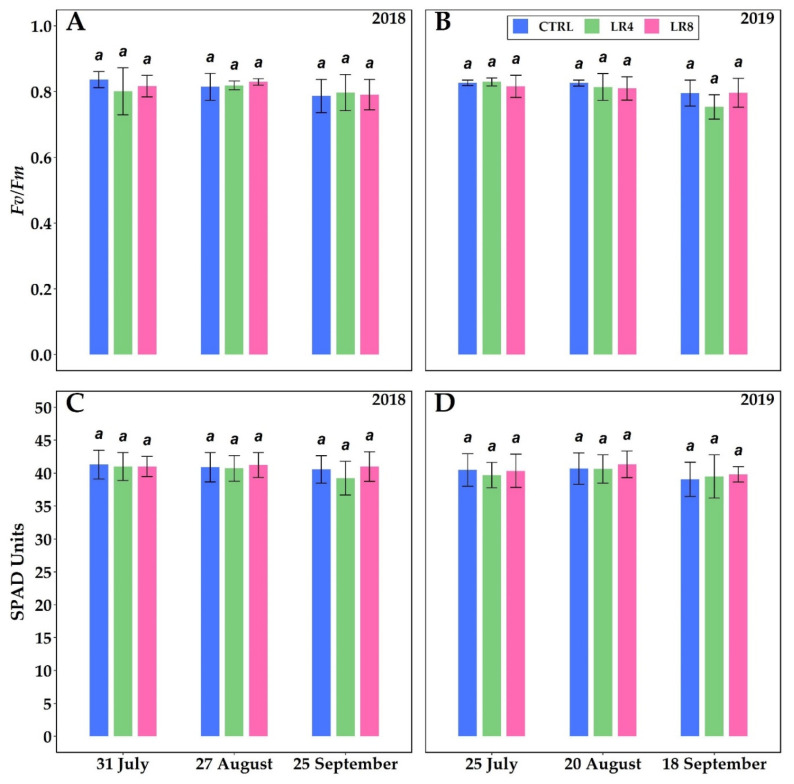
Maximum quantum yield of PSII (F_v_/F_m_) ((**A**), 2018; (**B**), 2019) and chlorophyll content (SPAD Units) ((**C**), 2018; (**D**), 2019) in *V. vinifera* with three different defoliation management methods: four leaf removal (LR4, green column), eight leaf removal (LR8, pink column), and no defoliation (CTRL, blue column). Measurements were conducted at full veraison (31 July 2018 and 25 July 2019), mid-maturation (27 August 2018 and 20 August 2019) and full maturation (25 September 2018 and 18 September 2019). Different letters within the same parameter indicate significant differences. Data (mean ± SE, n = 10) were subjected to one-way ANOVA (LSD test, *p* ≤ 0.05).

No significant differences were noted in chlorophyll a fluorescence (F_v_/F_m_) and chlorophyll content in leaves of *V. vinifera* as reported in Figure 2A–D.

The physiological parameters (leaf gas exchanges) of *V. vinifera* in the three different canopy management methods (four leaf removal LR4, eight leaf removal LR8, and no defoliation CTRL) are presented in Table 2.

No significant differences in physiological parameters (P_n_, g_s_) between LR4 and LR8 during the two study seasons (2018 and 2019) were found; CTRL was significantly different from the other two treatments. No significant differences in leaf water potential among leaf removal treatments were found. Leaf water potential values reflect seasonal trends; peaks of increased water stress were recorded in August, the driest and hottest month. No significant differences in *eWUE* among treatments were found at the mid-maturation stage (27 August 2018 and 20 August 2019).

### 2.3. Berry Composition and Temperature

Table 3 and Table 4 show the composition of *V. vinifera* berries under three different canopy management approaches in the two study years in terms of technological and phenolic maturities.

No significant difference in pH was found at full veraison and mid-maturation in both seasons, while a significant difference in pH was found at harvest: the LR8 treatment showed the lowest values (3.22 in 2018 and 3.05 in 2019). Consequently, LR8 was given the highest total acidity values during full maturation in both seasons (6.41 mg L^−1^ and 6.02 mg L^−1^, respectively). Regarding sugar content values, significant differences at harvest were noted in both seasons: LR4 and CTRL had the highest values (26.88 and 26.16 °Brix in 2018 and 27.75 and 26.90 °Brix in 2019, respectively). 

The greatest differences were found with regard to the composition of extractable anthocyanins in both seasons. At full veraison, mid- and full maturation, LR4 berries showed significantly higher extractable anthocyanin content compared to LR8 and CTRL berries in 2018 and 2019. The lowest values were recorded for the LR8 treatment at the three different stages: 344.15, 595.00, and 645.25 mg L^−1^ during the 2018 season and 505.34, 1025.31, and 695.37 mg L^−1^ during the 2019 season. At full maturation, LR4 berries showed significantly higher extractable polyphenol content compared to the other two treatments. No differences in total polyphenols at harvest were found.

**Figure 3:**
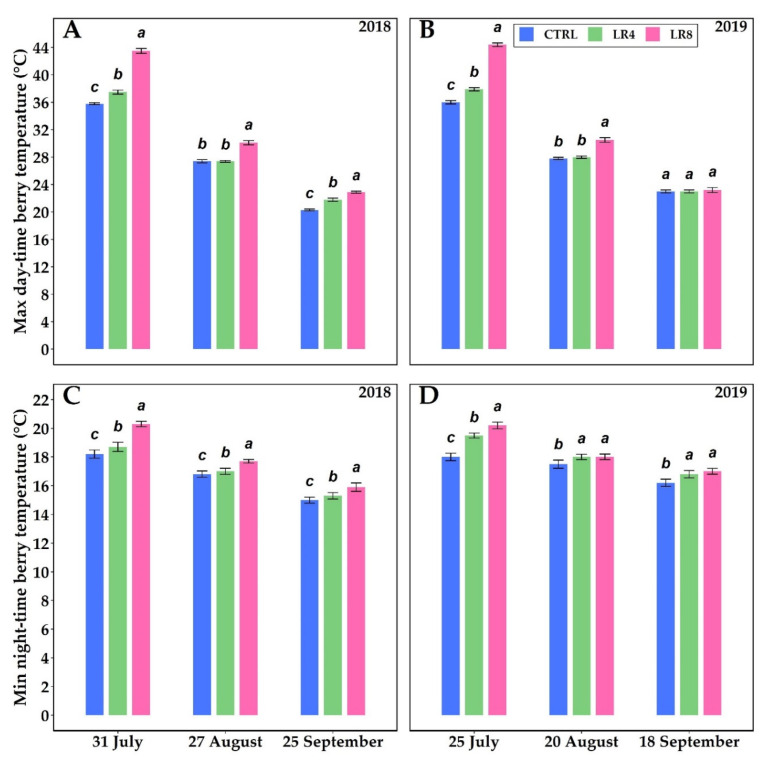
Maximum daytime temperature (°C) ((**A**), 2018; (**B**), 2019) and minimum night-time temperature (°C) ((**C**), 2018; (**D**), 2019) of *V. vinifera* berries with three different defoliation managements: four leaf removal (LR4, green column), eight leaf removal (LR8, pink column), and no defoliation (CTRL, blue column). Measurements were conducted at full veraison (31 July 2018 and 25 July 2019), mid-maturation (27 August 2018 and 20 August 2019) and full maturation (25 September 2018 and 18 September 2019). Different letters within the same parameter indicate significant differences. Data (mean ± SE, n = 10) were subjected to one-way ANOVA (LSD test, *p* ≤ 0.05).

Figure 3 shows berry temperature (°C) of *V. vinifera* among the three different canopy management approaches during the study period for the two years.

The maximum daytime and minimum night-time temperatures of berries at three different phenological phases were investigated (Figure 3). Temperature decreased from July until September when it dropped steadily. The highest daytime temperature values were recorded in the LR8 treatment, reaching 43.5 °C and 44.4 °C on 31 July 2018 and 25 July 2019, respectively. The same trend was observed for night-time temperature. The CTRL treatment gave the lowest values during the 2018 and 2019 seasons.

**Figure 4:**
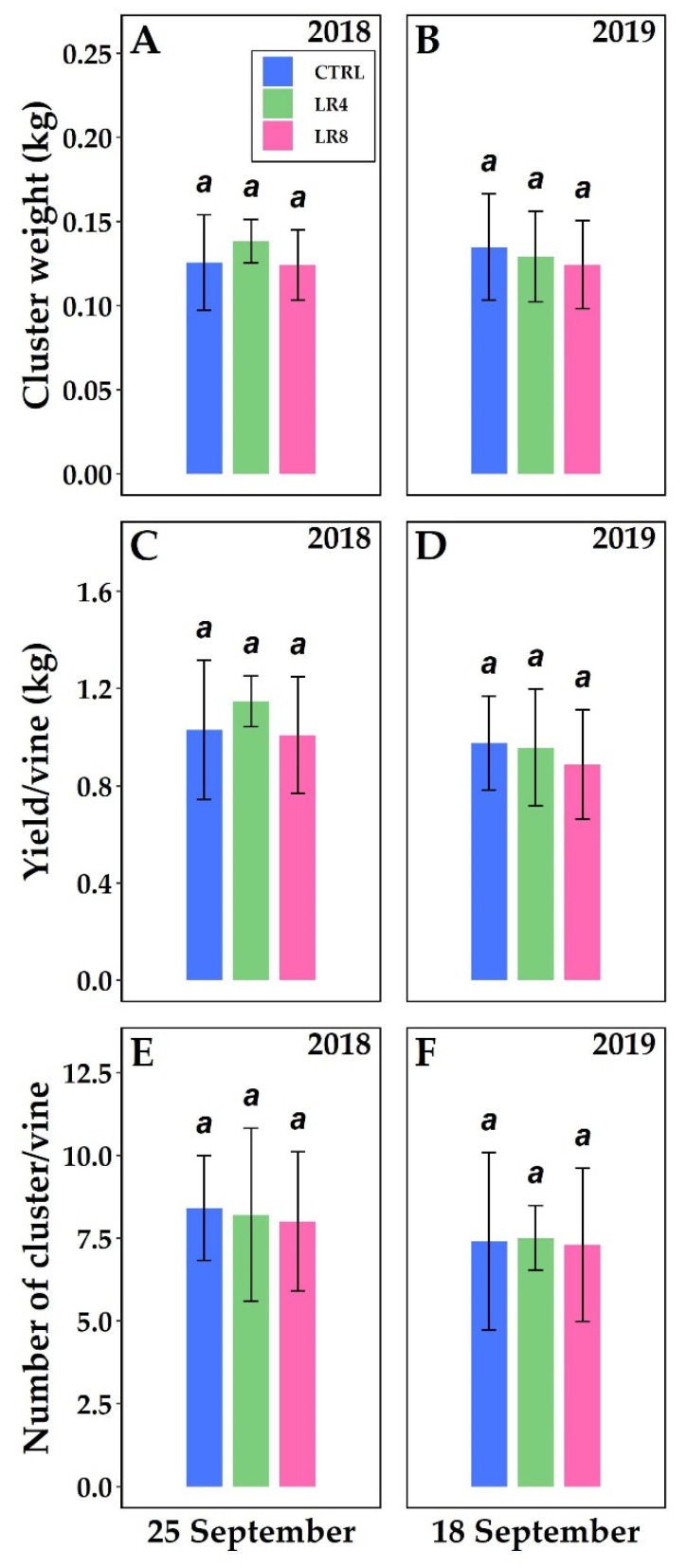
Cluster weight (kg) ((**A**), 2018; (**B**), 2019), yield/vine (kg) ((**C**), 2018; (**D**), 2019), and number of cluster/vine ((**E**), 2018; (**F**), 2019) of *V. vinifera* with three different defoliation management: four leaf removal (LR4, green column), eight leaf removal (LR8, pink column), and no defoliation (CTRL, blue column). Measurements were conducted at full maturation (25 September 2018 and 18 September 2019). Different letters within the same parameter indicate significant differences. Data (mean ± SE, n = 10) were subjected to one-way ANOVA (LSD test, *p* ≤ 0.05).

Figure 4 shows production data of *V. vinifera* among three different canopy management, under two years. No significant differences were found.

## 3. Discussion

The principal objective of canopy management is to maintain an environment that encourages suitable vegetative growth of vines and safeguards future clusters [45]. Practices include shoot trimming, vigor control, and leaf removal in the fruit zone [46,47].

This study assesses the importance of canopy management in the Mediterranean area through leaf removal as a potential tool to improve Cabernet Sauvignon grape quality.

Defoliation reduced the leaf area of the vines in this study by approximately 25% in the LR4 and 45% in the LR8 treatment. Considering a single leaf, net photosynthesis (P_n_) was lower in CTRL at veraison, mid-maturation, and full maturation. Defoliation increases single leaf photosynthesis [48]. However, in a LR8 treatment, leaf removal from the lower quarter of the canopy during berry growth can cause a significant decrease in whole-vine photosynthesis, thus suggesting that higher specific photosynthesis is not able to compensate the reduction in total leaf area [49,50]. Therefore, it is probable that leaf thinning reduces the potential CO_2_ assimilating surface, which potentially decreases total photosynthetic rates and influences the vine’s carbon balance [51]. Instead, it has been hypothesized that in the LR4 treatment, there were no changes in whole-vine photosynthesis (a vine maintained its photosynthesis rate even though 27% of the leaf area had been removed [50]). Generally, the rate of photosynthesis declines as the season progresses [52,53,54,55]. Unlike other plant species, a minimally reduced source-sink ratio resulted in no increased stomatal conductance, as well as no higher photosystem II (PSII) efficiency [56]. 

In both years studied, stomatal conductance, even in the hottest period, maintained optimal values: Cabernet Sauvignon reflects its anisohydric-conservative behavior under stress conditions without showing a drop in values [57]. Although water stress threshold values were found in August (in 2018: −1.27, −1.28, −1.36 MPa and in 2019: −1.28, −1.30, −1.37 MPa) [58], no significant differences were recorded in terms of leaf water potential.

Fluorescence of chlorophyll a (an indicator of photo-oxidative stress [59]) leads to us saying in our experiment that in both years, there was neither damage to PSII nor even excessively limiting situations for plants as no significant differences were seen in the two vintages. 

In our study, berry temperatures were influenced by defoliation. An increase in sun exposure leads to temperature elevation and fully exposed clusters are subjected to more pronounced fluctuations in daytime and night-time temperatures [60]. In July 2018, the LR4 treatment presented a daytime berry temperature that was 4.75% higher than CTRL; LR8 was 24.35% higher. In 2019, LR4 daytime berry temperature was 5.50% higher than CTRL; LR8 was 23.70% higher. The timing of fruit set basal defoliation has a stronger impact on the source-sink balance and light and temperature exposure than veraison defoliation [61].

In accordance with Zoecklein et al. [25], drastic leaf removal at harvest (25 September 2018 and 18 September 2019) reduced fruit soluble solids concentration: the sugar content of LR8 was lower than the other two treatments. Depending on the growth environment, values of between 7 and 14 cm^2^ of leaf area per gram of berry are often quoted as being necessary to satisfactorily ripen the fruit [62]. In fact, a drastic reduction in leaf area due to leaf removal could negatively affect the carbon balance and allocation to sinks as well as reproductive activity which may, in turn, negatively affect plant carbohydrate reserve replenishment [63,64]. On the other hand, partial defoliation (LR4) can improve vine performance [65]: the LR4 treatment presented the highest sugar content during the 2019 season.

As pointed out by Main et al. [66], leaf removal treatments had no effect on berry weight.

Leaf removal (LR4 treatment) generally increased fruit titratable acidity (TA), which was associated with a greater malic acid concentration [67].

With regard to the total and extractable anthocyanins and extractable polyphenols at harvest, the LR4 treatment gave the highest values. An increase in grape flavonol and anthocyanin concentration as an outcome of defoliation is due to sunlight-driven upregulation biosynthesis of polyphenols [68]. While the LR8 treatment had the lowest accumulation of both anthocyanins and polyphenols, this was probably due to excess sun exposure and excess temperature. In fact, some studies have reported that high levels of light and temperature have led to a decrease in anthocyanin levels [69]. It was highlighted that the lower anthocyanin content in berries under high temperature reflects the impact of reduced biosynthesis with an increased degradation (peroxidase enzymes in anthocyanin catabolism) [70]. The higher anthocyanin content in our LR4 treatment could also be due to a greater sugar content, which could, in turn, lead to an accumulation of this flavonoid [71].

## 4. Materials and Methods

### 4.1. Experimental Design and Settings

The experiment was carried out in a *Vitis vinifera* L. (cv. Cabernet Sauvignon, clonal selection 685) vineyard situated on a sandy soil in Bolgheri, Castagneto Carducci, Tuscany (43°23′39″ N; 10°61′6″ E) with an elevation of 96 m a.s.l. with south–west exposure. Soil horizons present a clay texture with the following characteristics: sand 40.1%; silt 32.5%; clay 27.4%; organic matter 1.3%; pH (H_2_O) 7.6. The experiment was set up during two consecutive seasons (2018 and 2019). The vineyard was planted in 2009 on 1103 P rootstock at a spacing of 1.1 by 2 m (∼4545 vines/ha) and trained on a vertical shoot positioning system in SE-NW oriented rows with a single cordon system, at 70 cm above ground with six spurs (12 buds per vine). The vines do not have irrigation and cultural practices were the common ones in the area. Pest treatments were applied as per local practices; no sprays against Botrytis were performed (Tuscan integrated management). 

The following three management methods were tested: removal of the first four leaves of each primary shoot (LR4), removal of the first eight leaves of each primary shoot (LR8), and no defoliation treatment (CTRL). Treatments were performed at the same time during the season, at fruit set, right after the machine shoot trimming. Treatments were applied according to a randomized block design with 10 replications per treatment. A single span, composed of five plants, was considered as a replication. Leaf removal was carried out on 18 June 2018 and on 12 June 2019. As in Mucalo’s experiment [72], in the defoliated nodes, lateral shoots were removed (they experience the highest photosynthetic activity during ripening and can cause a carry-off effect). Maximum, minimum, mean air temperatures (°C) and global radiation (W m^−2^) of the growing season (2018–2019) were collected daily from a weather station located in the vineyard (Ecotech, Germany); rainfall and air humidity were also recorded. As described thereafter, eco-physiological measurements were performed on 10 vines/management method (10 vines per treatment; one vine per replication) at three different dates corresponding to three different phenological stages: 31 July 2018 and 25 July 2019 (full veraison, 100% of berries present full color change; E-L 36 stage); 27 August 2018 and 20 August 2019 (mid-maturation; E-L 37 stage); 25 September 2018 and 18 September 2019 (full maturation; E-L 38 stage) [73].

### 4.2. Leaf Gas Exchanges, Leaf Area, Leaf Water Potential, Leaf Chlorophyll a Fluorescence and Content

Leaf gas exchange (transpiration rate (E), net photosynthesis (P_n_), and stomatal conductance (*g_s_*)) was measured using a portable infrared gas analyzer (model Ciras 3, PP Systems, Amesbury, MA, USA), between 10 and 12 a.m. on 10 fully developed and healthy leaves per treatment (10 replicates, one each vine). The photosynthesis/transpiration ratio, extrinsic water use efficiency (eWUE), was calculated. Measurements were performed, setting the leaf chamber flow under the same conditions as the experiment of [74]: saturating photosynthetic photon flux of 1300 μmol m^−2^s^−1^, ambient CO_2_ concentration ~400 ppm, and ambient temperature) at the three phenological stages as mentioned above. Based on the correlation between the vein length on the lower surface and leaf area, the leaf area of total assimilation area per vine was established by a non-destructive method [75]. Total leaf area was calculated by multiple regression analysis based on the following variables: leaf number, shoot length, and area of the smallest and largest leaf; in addition, the total leaf area was established using the number of shoots per plant [76]. 

Leaf water potential (Ψ_leaf_, MPa) was determined using a pressure chamber (model 600, PMS Instrument Co., Albany, OR, USA) on 10 fully expanded leaves per treatment, at approximately 12 noon–1:00 p.m. [77]. 

Using a chlorophyll fluorometer (Handy-PEA^®^, Hansatech Instruments, Norfolk, UK), the fluorescence of chlorophyll a (Chl-a) was recorded; the leaves were adapted to the dark for 30 min with the help of leaf clips. F_v_/F_m_ (variable/maximal Chl-a fluorescence, the maximum quantum yield of photosystem PSII) was calculated following Maxwell and Johnson’s methodology [78] and were collected by applying a saturating flash of actinic light at 3000 μmol photons m^−2^s^−1^ for 1 s. 

A 502 SPAD device (Konica Minolta Inc., Japan) was used to measure chlorophyll a content in leaves. At the same three stages, Chl-a content, leaf water potential, and Chl-a fluorescence were measured on the same leaves used for leaf gas exchange measurements.

### 4.3. Berry Composition and Temperature

Choosing 20 sample berries per plant, 100 berries per replication were randomly harvested at full veraison, mid-maturation and full maturation from each treatment to perform technological maturity assessments. With a digital scale (model ES2201, Artiglass, Due Carrare, PD, Italy), each sample was individually weighed and the berries were then squeezed to for pH, sugar content, and titratable acidity (TA) analyses. A refractometer (PCE-Oe Inst., Lucca, Italy) was used to evaluate sugar content (°Brix). A portable pH meter (PCE-Oe Inst., Lucca, Italy) was used to evaluate pH of grape must, and, using 0.1 M NaOH to an end-point of pH 7.0, g L^−1^ tartaric acid (TA) was calculated by manual glass burette on a 10 mL sample. To determine the optimum harvesting time, berries from three different experimental plots were tested every other day with a digital hand refractometer and harvesting time was considered optimal when the sugar content in all treatments reached 25 °Brix. In addition, another 100-berry sample/vine/thesis (10 berry samples per plant) was used to determine phenolic contents as well as extractable and total anthocyanins [79]. During the three phenological phases, the daytime and night-time temperatures (12–1 p.m. and 2–3 a.m., respectively) of the bunch were measured by temperature probes (micro temperature probe—GMR_MTP) inserted inside the berries (GMR Strumenti SAS, Scandicci, Italy) [80,81]. A datalogger was connected to the probes of the single cluster. 

### 4.4. Statistical Analysis

Data from each season (2018–2019) were separately analyzed by means of one-way ANOVA with defoliation treatments as the main factor (*p* ≤ 0.05). In addition, mean values were separated by Fisher’s least significant difference (LSD) post hoc test (*p* ≤ 0.05). *p* value adjustment was performed with the Holm method. All statistical analyses were performed using R and RStudio (Boston, MA, USA) [82]. 

## 5. Conclusions

The present research investigated the influence of basal leaf removal on vine physiology, berry temperature, and grape quality based on three methods (removal of four leaves, LR4; eight leaves, LR8; and no defoliation, CTRL). Under defoliation conditions, considering the single leaf, vines treated with LR4 and LR8 maintained higher photosynthesis levels compared to CTRL. In terms of berry traits, higher sugar and anthocyanins content were detected in LR4 berries. These effects may make LR4 treatment a candidate as a practical tool for winemakers who want to increase vine performance. Overall, LR8 vines presented a slow maturation with unchanged berry weight and higher titratable acidity in comparison with LR4 and CTRL vines; this method could be useful for rosé wine production. Furthermore, the sharp decrease in anthocyanin concentration under severe defoliation suggests that the influence of light and temperature are dominant in the degradation of these compounds. To fully validate and clarify the effects of leaf removal on secondary metabolites (amount and extraction), further investigations are needed. Investigation of the processes of assimilation of CO_2_ at the level of the whole vine would also be useful.

## Figures and Tables

**Table 1:** Total leaf area (m^2^). Total leaf area (m^2^) of *V. vinifera* treated with three different defoliation treatments: four leaf removal (LR4), eight leaf removal (LR8), and no defoliation (CTRL), during two seasons (2018–2019). Measurements were conducted at four times: fruit set (18 June 2018 and 12 June 2019), full veraison (31 July 2018 and 25 July 2019), mid-maturation (27 August 2018 and 20 August 2019) and full maturation (25 September 2018 and 18 September 2019). Data (mean ± SE, n = 10) were subjected to one-way ANOVA. Different letters within the same parameter and row indicate significant differences (LSD test, *p* ≤ 0.05).

	Total Leaf Area (2018)			Total Leaf Area (2019)		
Stage	CTRL	LR4	LR8	CTRL	LR4	LR8
Fruit set	1.15 ± 0.46 a	0.77 ± 0.21 b	0.46 ± 0.12 c	1.00 ± 0.38 a	0.77 ± 0.17 b	0.42 ± 0.10 c
Full veraison	1.24 ± 0.40 a	0.82 ± 0.12 b	0.58 ± 0.21 b	1.11 ± 0.32 a	0.86 ± 0.11 b	0.54 ± 0.17 c
Mid-maturation	1.41 ± 0.49 a	0.99 ± 0.28 b	0.87 ± 0.34 b	1.31 ± 0.26 a	1.00 ± 0.31 a	0.74 ± 0.23 b
Full maturation	1.62 ± 0.34 a	1.19 ± 0.43 b	0.78 ± 0.35 c	1.55 ± 0.30 a	1.13 ± 0.32 ab	0.82 ± 0.28 b

**Table 2:** Physiological parameters. Net photosynthesis (*P_n_*), stomatal conductance (*g_s_*), water use efficiency (*eWUE*) and leaf water potential (Ψ_leaf_) of *V. vinifera* treated with three different defoliation management methods: four leaf removal (LR4), eight leaf removal (LR8), and no defoliation (CTRL). Measurements were conducted at full veraison, mid-maturation and full maturation. Data (mean ± SE, n = 10) were subjected to one-way ANOVA. Different letters within the same parameter and row indicate significant differences (LSD test, *p* ≤ 0.05).

	***P_n_* (µmol CO_2_ m^2^ s^−1^)**			***g_s_* (mmol H_2_O m^2^ s^−1^)**		
**Stage**	**CTRL**	**LR4**	**LR8**	**CTRL**	**LR4**	**LR8**
31 July 2018	11.05 ± 4.11 b	14.68 ± 3.96 a	15.11 ± 2.87 a	126.50 ± 41.78 b	198.00 ± 31.63 a	187.70 ± 24.43 a
27 August 2018	8.24 ± 2.08 b	12.51 ± 2.62 a	13.85 ± 3.85 a	114.87 ± 16.08 a	117.70 ± 26.43 a	115.35 ± 32.73 a
25 September 2018	7.98 ± 1.99 b	9.46 ± 2.76 a	9.16 ± 3.80 a	99.49 ± 22.45 b	120.11 ± 34.17 a	124.10 ± 24.53 a
25 July 2019	10.65 ± 3.42 b	15.42 ± 1.58 a	15.55 ± 1.57 a	116.25 ± 35.66 b	145.10 ± 33.85 b	209.80 ± 29.30 a
20 August 2019	7.58 ± 2.68 b	11.93 ± 2.56 a	12.53 ± 3.37 a	99.60 ± 20.41 b	115.55 ± 32.17 a	102.88 ± 29.15 ab
18 September 2019	5.82 ± 2.33 b	7.61 ± 2.56 a	7.64 ± 3.75 a	92.30 ± 22.70 a	91.40 ± 31.75 a	95.65 ± 33.08 a
	***eWUE* (µmol CO_2_/mmol H_2_O)**			***Ψ_leaf_* (MPa)**		
**Stage**	**CTRL**	**LR4**	**LR8**	**CTRL**	**LR4**	**LR8**
31 July 2018	2.64 ± 0.54 b	2.26 ± 0.31 ab	1.58 ± 0.60 a	−1.04 ± 1.41 a	−1.07 ± 1.22 a	−1.03 ± 1.39 a
27 August 2018	2.00 ± 0.37 a	1.82 ± 0.64 a	1.69 ± 0.68 a	−1.27 ± 1.71 a	−1.28 ± 1.81 a	−1.36 ± 0.99 a
25 September 2018	2.89 ± 0.32 b	2.02 ± 0.55 a	2.11 ± 0.84 a	−1.25 ± 1.36 a	−1.26 ± 1.52 a	−1.20 ± 1.33 a
25 July 2019	2.76 ± 0.78 b	1.70 ± 0.82 a	1.68 ± 0.46 a	−1.07 ± 0.70 a	−1.05 ± 1.08 a	−1.06 ± 0.98 a
20 August 2019	1.65 ± 0.54 a	1.49 ± 0.23 a	1.45 ± 0.46 a	−1.28 ± 1.79 a	−1.30 ± 1.95 a	−1.37 ± 1.13 a
18 September 2019	2.53 ± 0.75 b	1.77 ± 0.83 ab	1.41 ± 0.59 a	−1.29 ± 1.77 a	−1.29 ± 1.61 a	−1.21 ± 1.68 a

**Table 3:** Technological maturity. Sugar content (°Brix), titratable acidity (TA), pH and berry weight of *V. vinifera* treated with three different defoliation managements: four leaf removal (LR4), eight leaf removal (LR8), and no defoliation (CTRL). Measurements were conducted at full veraison (31 July 2018 and 25 July 2019), mid-maturation (27 August 2018 and 20 August 2019) and full maturation (25 September 2018 and 18 September 2019). Data (mean ± SE, n = 10) were subjected to one-way ANOVA. Different letters within the same parameter and row indicate significant differences (LSD test, *p* ≤ 0.05).

	**Sugar Content (°Brix)**			**TA (mg L^−1^ Tartaric Acid)**		
**Stage**	**CTRL**	**LR4**	**LR8**	**CTRL**	**LR4**	**LR8**
31 July 2018	12.92 ± 0.09 a	13.81 ± 0.28 a	12.7 ± 0.35 a	13.84 ± 0.07 a	13.68 ± 0.05 a	14.52 ± 0.09 a
27 August 2018	22.00 ± 0.12 a	21.89 ± 0.18 a	20.81 ± 0.20 a	6.90 ± 0.10 b	7.30 ± 0.07 ab	7.74 ± 0.03 a
25 September 2018	26.16 ± 0.09 a	26.88 ± 0.15 a	25.19 ± 0.05 b	5.98 ± 0.03 ab	5.86 ± 0.04 b	6.41 ± 0.06 a
25 July 2019	13.10 ± 0.06 b	16.29 ± 0.10 a	13.08 ± 0.07 b	13.42 ± 0.08 a	13.00 ± 0.06 a	13.82 ± 0.07 a
20 August 2019	22.50 ± 0.07 a	22.59 ± 0.03 a	21.26 ± 0.15 a	6.36 ± 0.04 b	6.70 ± 0.08 ab	7.54 ± 0.010 a
18 September 2019	26.90 ± 0.22 b	27.75 ± 0.21 a	25.98 ± 0.19 c	5.43 ± 0.07 b	5.53 ± 0.09 b	6.02 ± 0.06 a
	**pH**			**Berry Weight (g)**		
**Stage**	**CTRL**	**LR4**	**LR8**	**CTRL**	**LR4**	**LR8**
31 July 2018	2.60 ± 0.05 a	2.75 ± 0.02 a	2.55 ± 0.05 a	0.88 ± 0.02 a	0.81 ± 0.04 a	0.82 ± 0.02 a
27 August 2018	3.28 ± 0.08 a	3.22 ± 0.04 a	3.10 ± 0.05 a	1.12 ± 0.10 a	1.00 ± 0.08 ab	0.90 ± 0.05 b
25 September 2018	3.53 ± 0.03 a	3.54 ± 0.02 a	3.22 ± 0.04 b	1.18 ± 0.05 a	1.16 ± 0.02 a	1.13 ± 0.03 a
25 July 2019	3.04 ± 0.08 a	3.02 ± 0.03 a	3.02 ± 0.07 a	0.85 ± 0.04 a	0.75 ± 0.03 a	0.70 ± 0.04 b
20 August 2019	3.30 ± 0.02 a	3.28 ± 0.03 a	3.21 ± 0.01 a	1.03 ± 0.05 a	0.92 ± 0.05 a	0.98 ± 0.03 a
18 September 2019	3.70 ± 0.05 a	3.64 ± 0.03 a	3.05 ± 0.02 b	1.15 ± 0.08 a	1.12 ± 0.05 a	1.12 ± 0.03 a

**Table 4:** Phenolic maturity. Total anthocyanin (Tot. Anth.), extractable anthocyanin (Extr. Anth.), total polyphenol (Tot. Polyp.), and extractable polyphenol (Extr. Polyp.) contents of *V. vinifera* treated with three different defoliation managements: four leaf removal (LR4), eight leaf removal (LR8), and no defoliation (CTRL). Measurements were conducted at full veraison (31 July 2018 and 25 July 2019), mid-maturation (27 August 2018 and 20 August 2019) and full maturation (25 September 2018 and 18 September 2019). Data (mean ± SE, n = 10) were subjected to one-way ANOVA. Different letters within the same parameter and row indicate significant differences (LSD test, *p* ≤ 0.05).

	**Tot. Anth. (mg L^−1^)**			**Extr. Anth. (mg L^−1^)**		
**Stage**	**CTRL**	**LR4**	**LR8**	**CTRL**	**LR4**	**LR8**
31 July 2018	751.41 ± 27.68 b	880.25 ± 16.44 a	738.50 ± 10.68 b	370.40 ± 9.21 b	393.73 ± 13.86 a	344.15 ± 9.41 c
27 August 2018	1450.75 ± 24.87 b	1550.50 ± 21.22 a	1312.50 ± 17.89 c	638.75 ± 15.87 b	653.75 ± 11.04 a	595.00 ± 14.16 c
25 September 18	1690.15 ± 23.55 b	1830.12 ± 19.48 a	1490.75 ± 14.32 c	737.15 ± 7.60 b	850.15 ± 10.09 a	645.25 ± 13.91 c
25 July 2019	938.23 ± 11.74 b	1030.20 ± 8.86 a	910.51 ± 7.53 b	561.22 ± 7.60 b	743.20 ± 15.58 a	505.34 ± 14.78 c
20 August 2019	1605.20 ± 10.00 b	2030.77 ± 12.62 a	1459.50 ± 12.62 c	1241.27 ± 9.44 b	1470.50 ± 7.66 a	1025.31 ± 11.73 c
18 September 19	1711.00 ± 23.14 b	2227.50 ± 7.08 a	1437.30 ± 21.98 c	782.51 ± 16.12 b	1087.78 ± 9.12 a	695.37 ± 10.07 c
	**Tot. Polyp. (mg L^−1^)**			**Extr. Polyp. (mg L^−1^)**		
**Stage**	**CTRL**	**LR4**	**LR8**	**CTRL**	**LR4**	**LR8**
31 July 2018	3164.54 ± 46.76 b	3247.10 ± 43.16 a	3195.27 ± 36.89 b	2832.80 ± 39.67 a	2889.67 ± 45.98 a	2876.90 ± 42.76 a
27 August 2018	3395.87 ± 31.72 b	3439.08 ± 33.05 ab	3457.15 ± 29.34 a	2920.60 ± 45.00 a	2974.97 ± 34.16 a	2901.56 ± 67.81 a
25 September 18	3367.29 ± 49.15 a	3155.70 ± 54.25 b	3389.34 ± 44.21 a	2889.45 ± 32.57 a	2950.12 ± 54.15 a	2745.98 ± 64.75 b
25 July 2019	4021.65 ± 24.43 a	4005.21 ± 37.78 a	4051.81 ± 37.60 a	3682.59 ± 29.62 a	3614.36 ± 55.08 a	3669.18 ± 63.12 a
20 August 2019	3996.14 ± 43.22 a	4013.25 ± 47.25 a	3848.61 ± 34.31 b	3600.45 ± 42.00 a	3609.67 ± 24.17 a	3565.45 ± 35.90 b
18 September 19	3576.17 ± 38.27 a	3520.16 ± 32.25 a	3598.28 ± 34.46 a	3369.11 ± 57.51 b	3481.57 ± 55.15 a	3306.21 ± 45.32 b

## Data Availability

Not applicable.
